# Supplementary material for: Controlled Growth of WO3 Photoanode under Various pH Conditions for Efficient Photoelectrochemical Performance
Source: Nanomaterials (Basel). 2023 Dec 19;14(1):8. doi: 10.3390/nano14010008 (PMC10780304; doi:10.3390/nano14010008)
Supplement: Supplementary file 1 [file nanomaterials-14-00008-s001.zip › nanomaterials-2760390-supplementary.pdf]

nanomaterials

Supplementary Materials

Controlled Growth of WO_3 Photoanode under Various pH Conditions for Efficient Photoelectrochemical Performance

Seung-Je Yoo, Dohyun Kim and Seong-Ho Baek *

Department of Energy Engineering, Dankook University, Cheonan 31116, Republic of Korea
* Correspondence: seongho@dankook.ac.kr

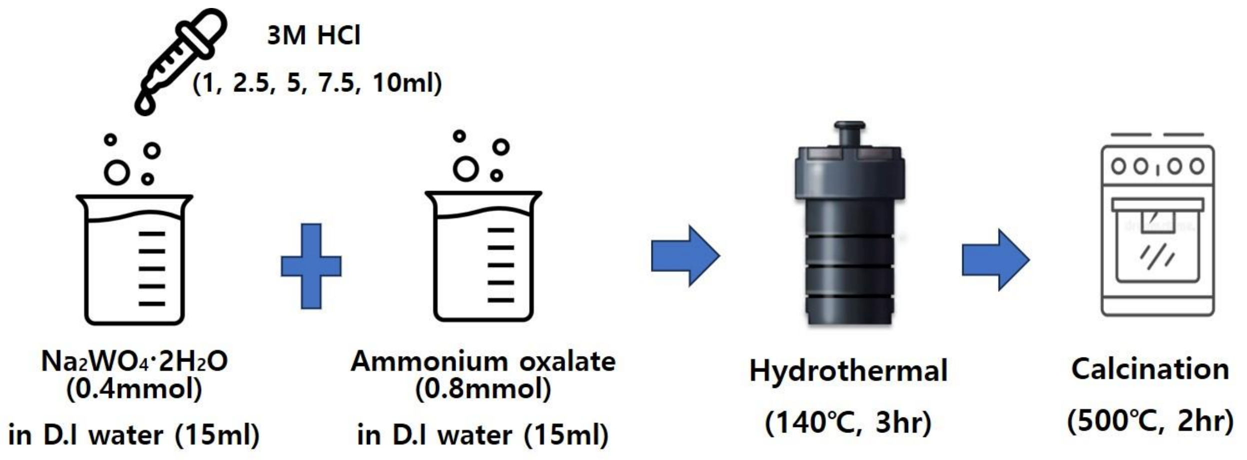

Figure S1. Schematics of the preparation procedures of $WO_3{\cdot}nH_2O$ and WO_3 materials.

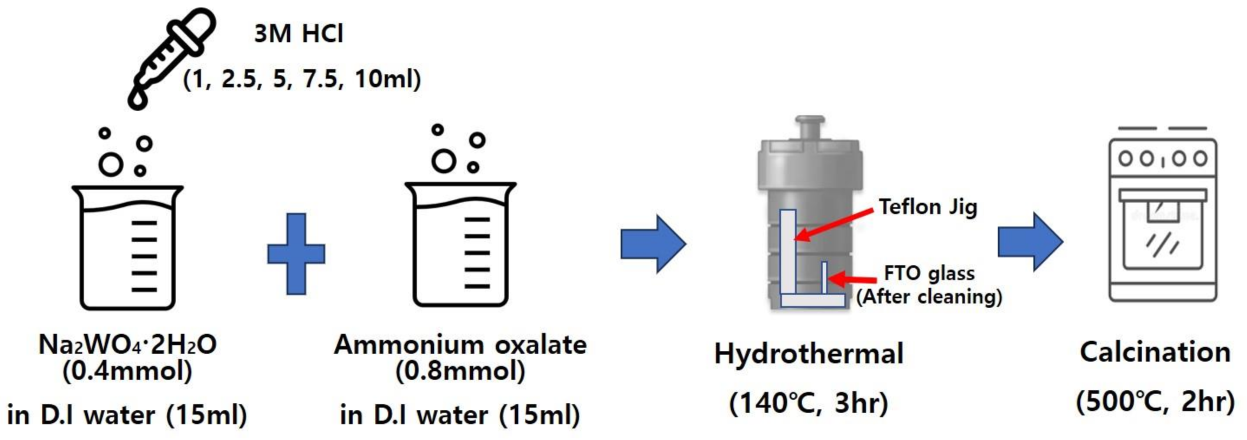

Figure S2. Illustrations of the preparation procedures of WO_3 photoanode on FTO substrate.

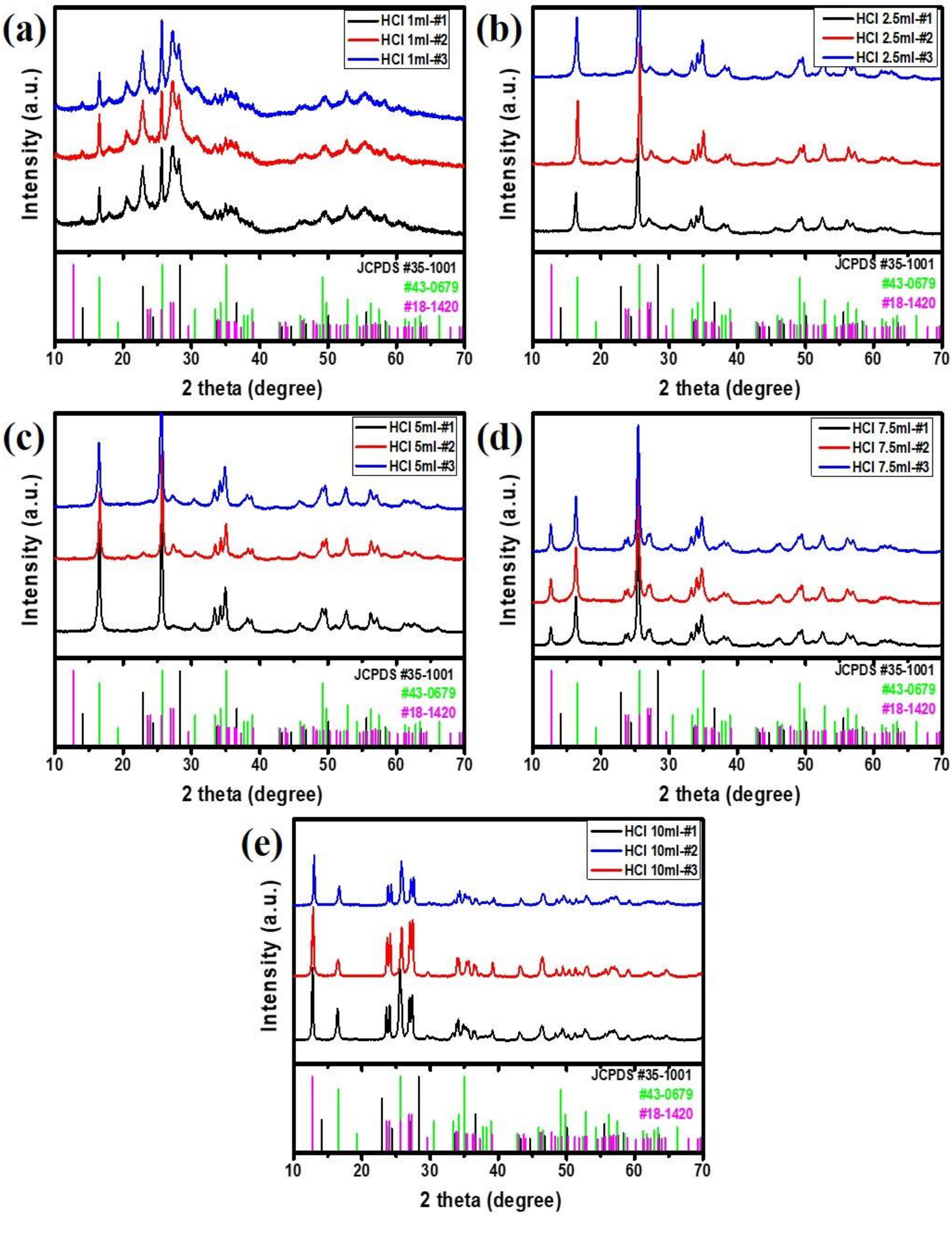

Figure S3. XRD spectra of the $WO_3 \cdot nH_2O$ powders obtained by acid precipitation process with 2theta range from 10° to 70°. (a) HCl-1ml, (b) HCl-2.5ml, (c) HCl-5ml, (d) HCl-7.5ml, (e) HCl-10ml samples.

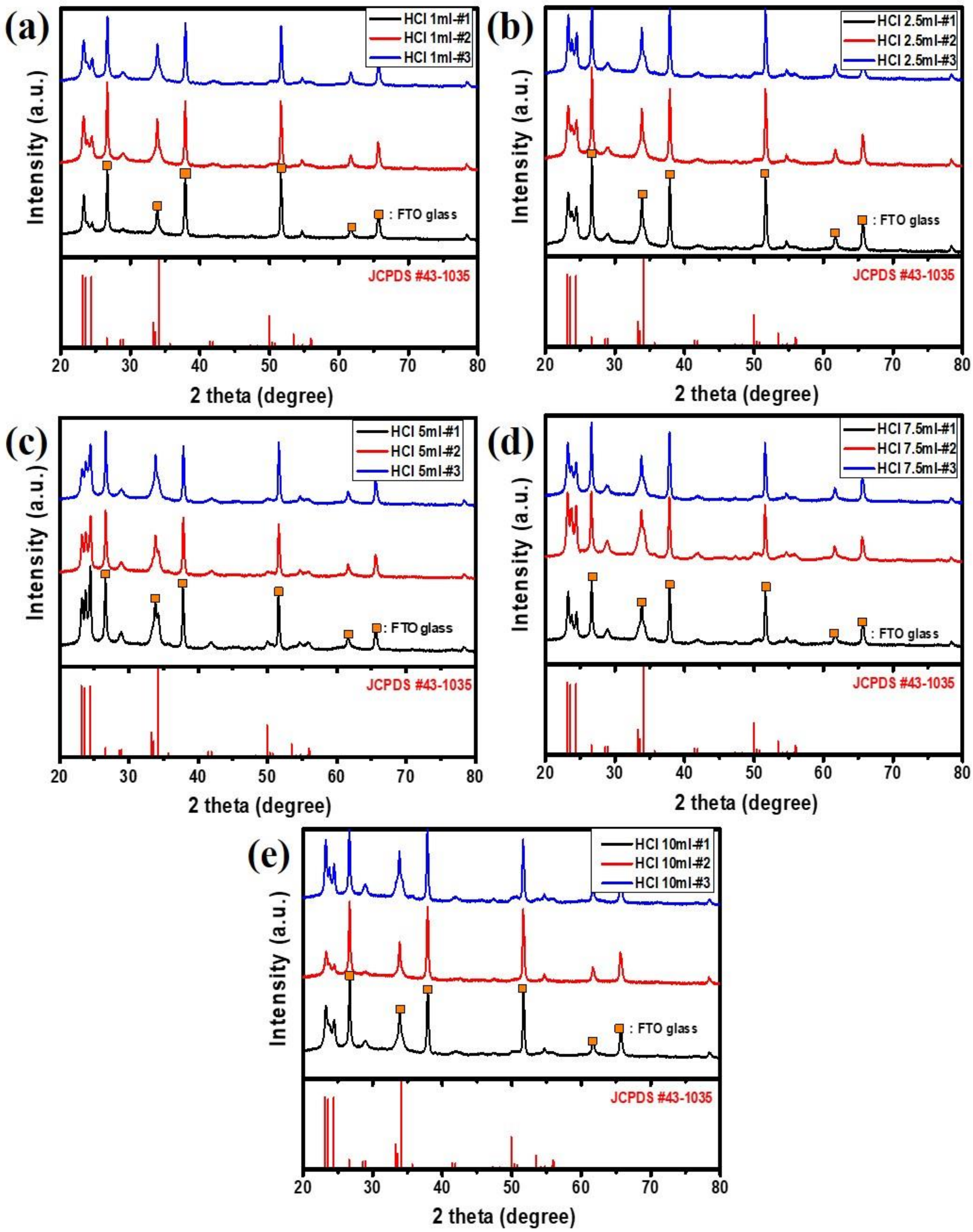

Figure S4. XRD spectra of the WO_3 nanoplates grown on FTO glass with different HCl volume. (a) HCl-1ml, (b) HCl-2.5ml, (c) HCl-5ml, (d) HCl-7.5ml, (e) HCl-10ml samples.

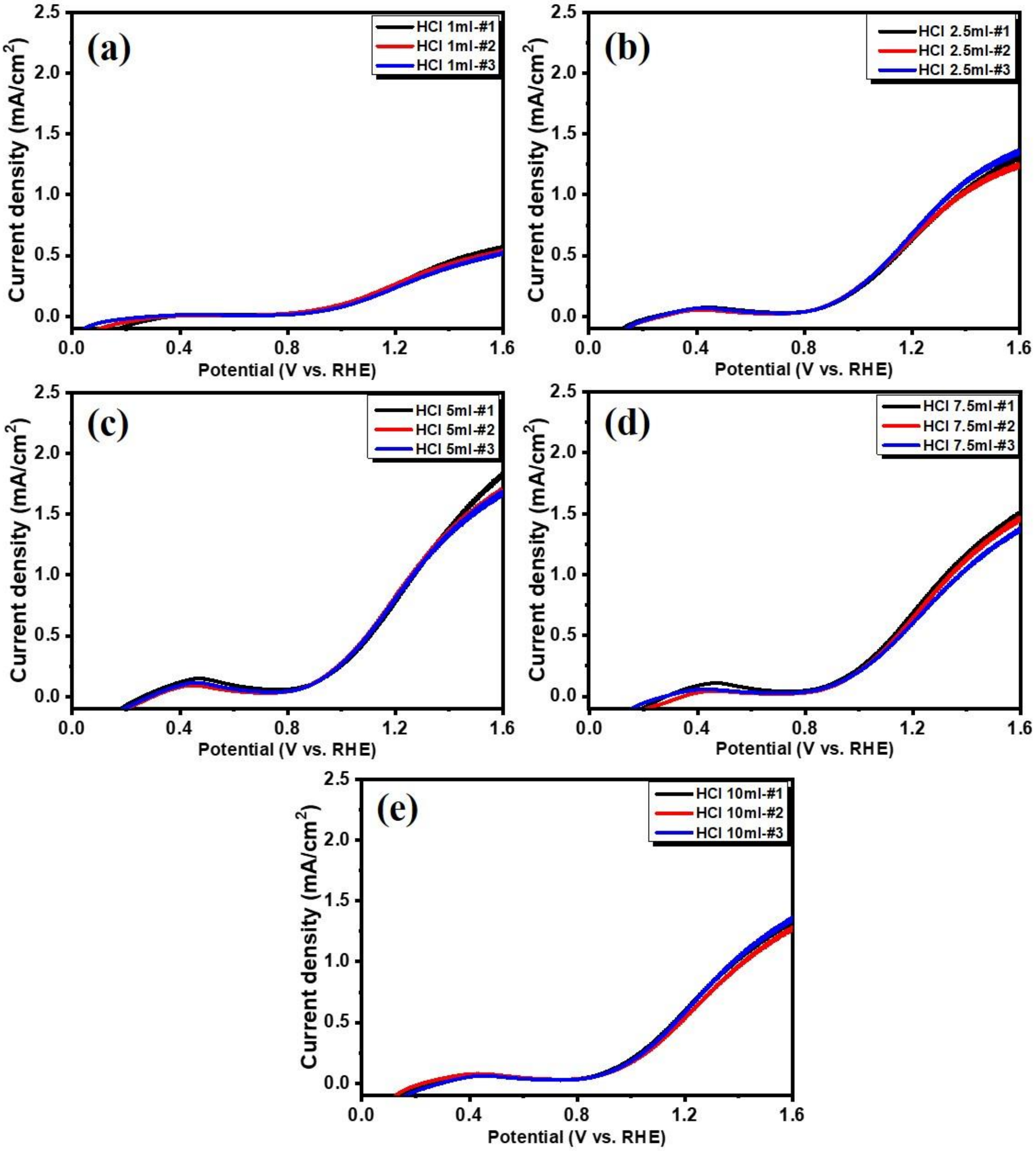

Figure S5. LSV curves obtained from the WO_3 photoanodes under AM 1.5G condition. (a) HCl-1ml, (b) HCl-2.5ml, (c) HCl-5ml, (d) HCl-7.5ml, (e) HCl-10ml samples.

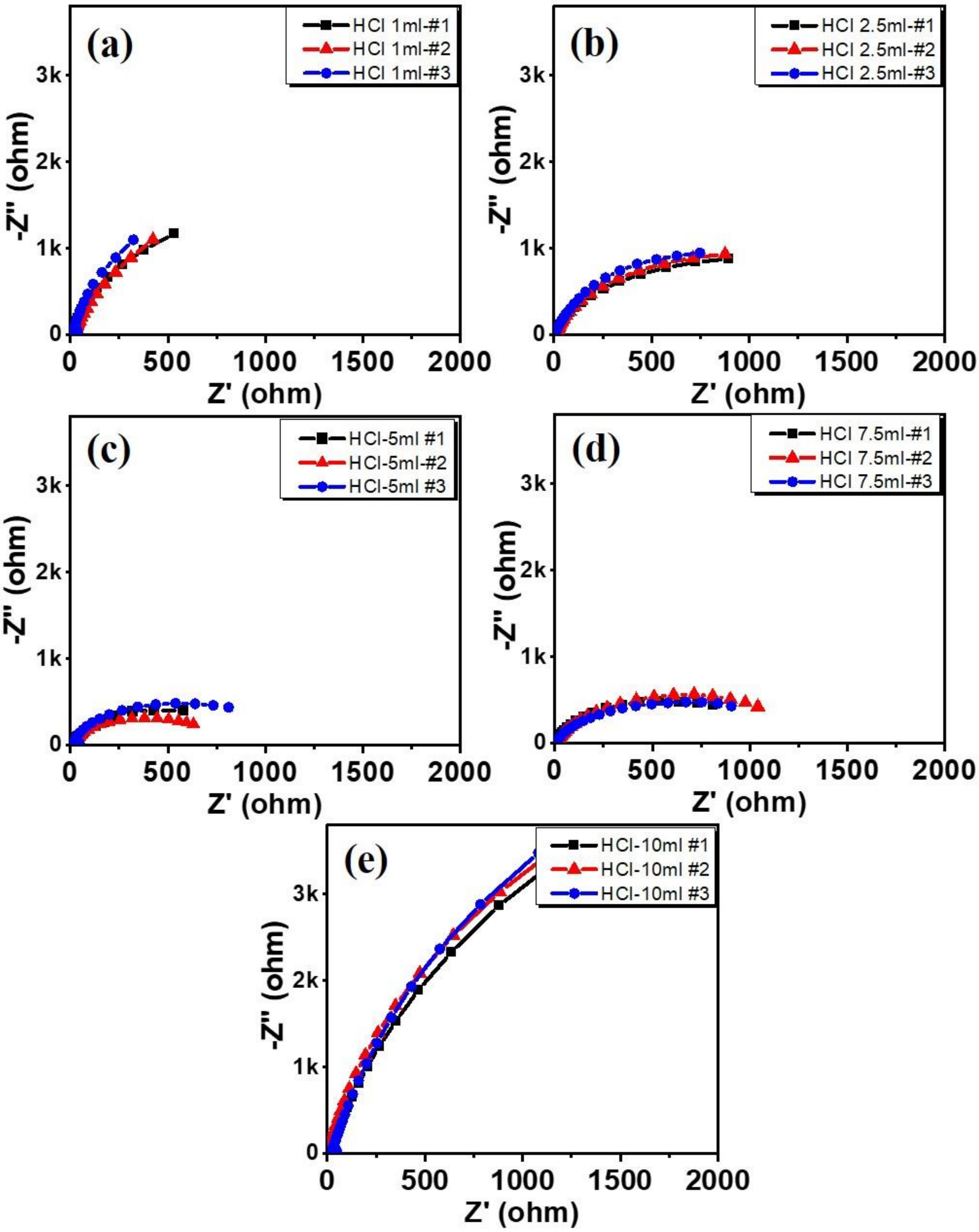

Figure S6. EIS Nyquist plots of all the photoanodes under AM 1.5G condition. (a) HCl-1ml, (b) HCl-2.5ml, (c) HCl-5ml, (d) HCl-7.5ml, (e) HCl-10ml samples.
